# Advances in Tourette’s syndrome

**DOI:** 10.1007/s00415-023-11588-3

**Published:** 2023-02-17

**Authors:** Neil P. Robertson

**Affiliations:** Institute of Psychological Medicine and Clinical Neuroscience, Cardiff University, University Hospital of Wales, Heath Park, Cardiff, CF14 4XN UK

## Introduction

Tourette’s is a neurodevelopmental disorder usually beginning in childhood or adolescence and presents with involuntary tics or vocalisations and is more common in males. It has an overall childhood prevalence of around 0.6% and has no ethnic or socio-economic predilection. Most affected children have another mental, behavioural, or developmental disorder with the commonest being anxiety and attention-deficit hyperactivity disorder and struggle with social competence and skills. Around one third of people with Tourette’s also have obsessive–compulsive disorder. Although in many cases tics decrease during adolescence and early adulthood, and indeed can disappear completely, some continue tics into adulthood and may even worsen. As a result, Tourette’s represents somewhat of a ‘Peter Pan’ disorder in that it commonly straddles care boundaries between child and adult neurology, psychiatric and psychology services.

There is currently no cure for the disorder although medications are commonly used to supress tics or treat associated conditions. A wide variety of drug interventions have been employed, some with limited evidence for efficacy, including antipsychotics, antiepileptics, tetrabenazine, antidepressants and even botulinum toxin injections, with many having potentially unacceptable short and long-term adverse effects. In addition, there is scant consensus on treatment or the order in which they are offered, with many decisions influenced by relevant individual factors. It is clear that new approaches are required and in this month’s journal club, we explore a meta-analysis of pharmacological intervention, a report of closed-loop deep brain stimulation technology and the use of exposure response prevention.

## Comparative efficacy, tolerability and acceptability of pharmacological interventions for the treatment of children, adolescents and young adults with Tourette’s syndrome: a systematic review and network meta-analysis

This systematic review and network analysis aimed to provide a more rigorous evidence base to guide management decisions and allow future guideline developments by comparing efficacy, tolerability and acceptability of drug treatments for Tourette’s. A comprehensive search of published and unpublished literature to November 20,231 was undertaken and included double-blind randomised trials of any medication administered as monotherapy in adults or children using standardised diagnostic criteria and excluded studies that exclusively recruited participants with attention-deficit hyperactivity disorder or obsessive–compulsive disorder. The Confidence in Network Meta-Analysis (CINeMA) framework was used to assess the certainty of evidence. Primary outcome was change in tic frequency. Summary data were extracted and pooled with a random-effects network meta-analysis to calculate standardised mean differences (SMD) and odds ratios with 95% confidence intervals.

Data from 4578 participants in 39 randomised controlled trials evaluating 23 medications across six medication categories were included in analysis. First (SMD − 0·65 [0·79 to − 0·51]) and second-generation antipsychotic drugs (SMD − 0·71 [− 0·88 to − 0·54]) as well as alpha-2 agonists (− 0·21 [− 0·39 to − 0·03]) were more efficacious than placebo. First- and second-generation antipsychotic drugs did not differ significantly from each other but outperformed alpha-2 agonists. Similar findings were observed for individual drugs but with aripiprazole and risperidone outperforming clonidine with moderate certainty. No relevant finding on tolerability or acceptability was identified for individual medications or against placebo.

*Comment* Whilst may large metanalyses such as these can be plagued by lack of detailed standardised data, this study explores an important issue in the management of Tourette’s and conclusions are appropriate and the authors are careful not to overstate the analysis. It also represents an important step in developing consensus guidelines for this condition.

Farhat LC, et al. Lancet Child Adolesc Health. 2022 Dec 14:S2352-4642(22)00,316–9. https://doi.org/10.1016/S2352-4642(22)00316-9. Epub ahead of print. PMID: 36528030.

## Embedded human closed-loop deep brain stimulation for Tourette syndrome: a nonrandomized controlled trial

Deep brain stimulation (DBS) is a treatment strategy for Tourette’s in individuals resistant to conventional pharmacological therapies and in whom the disorder is making a significant adverse impact. However, there are some disadvantages with conventional continuous stimulation and since Tourette’s is an episodic disorder, the authors of this study postulate that a closed-loop stimulator that is only activated when a previously identified physiological marker of tics is detected may be of advantage. In this non-randomised controlled trial primarily examining safety and feasibility, a bilateral centromedian parafascicular complex thalamic closed-loop DBS was implanted in six patients recruited and screened from a single centre. Results between closed and open loop stimulation was also compared and a patient-specific closed-loop paradigm was created for each patient. The primary outcome measure was a 40% reduction in the Yale Global Tic Severity Scale (YGTSS) at six months although there was also a comparison of closed and open loop stimulation using the Modified Rush Videotaping Scale for Tic (MRVRST). Two out of six patients were excluded as the closed-loop technology was not available to these patients at the time of implantation. In addition, one patient had not optimised closed-loop stimulation by six months so that outcome data were restricted to more short-term MRVRST.

Conventional DBS produced a 33.3% improvement on the YGTSS and 52.8% on the MRVRST and two patients had > 40% improvement at six months. No statistical differences in severity scores were observed between closed and open loop paradigms. The authors conclude that a patient-specific, closed-loop DBS was safe a feasible in patients with TS and may have some advantages over open loop systems.

*Comment* This very early report represents complex bioengineering technology integrated with neurosurgical expertise which is only likely to be applied to a small number of treatment resistant patients. There are clearly several limitations to the study including the small patient numbers, high number of adverse events, short battery life and lack of agreed minimally significant change in tic scales. However, the application of this patient-specific technology is groundbreaking and likely to provide some wider insights into Tourette’s over time.

Cagle JN, et al. JAMA Neurol. 2022 Oct 1;79(10):1064–1068. https://doi.org/10.1001/jamaneurol.2022.2741. PMID: 36094652; PMCID: PMC9468946.

## Therapist-supported online remote behavioural intervention for tics in children and adolescents in England (ORBIT): a multicentre, parallel group, single-blind, randomised controlled trial

Although adult neurologists are arguably more comfortable with the use of pharmacological interventions than behavioural therapies, they are more commonplace in paediatric management of tics and have demonstrated effectiveness. Exposure and response prevention (ERP) is one such behavioural therapy and in this study was delivered via internet, therapist supported, parent assisted to evaluate effectiveness for Tourette’s or chronic tic disorders.

This multicentre, parallel group, single-blind, randomised controlled trial was conducted across two study sites in England. Inclusion criteria were; aged 9–17, no behavioural therapy for 12 months with a YGTSS of > 15 or > 10 if they had only motor or vocal tics. Exclusion criteria included: change in medication for tics in the last 12 months, other significant mental health disorder, intellectual disability, inability to communicate in English. Patients were randomly assigned to receive 10 weeks of remotely delivered ERP or psychoeducation as an active control. Primary outcome was YGTSS three months after randomisation.

445 candidates were assessed, recruited from 16 identification sites. 221 were excluded and 224 enrolled and randomised. 11 patients in the ERP group and 11 in the psychoeducation group were lost to follow-up. Mean decrease in the YGTSS was 4.5 (16%) in the ERP and 1.6 (6%) in the active control group. The authors conclude that ERP is an effective behavioural therapy for tics in children and adolescents and offers an efficient and resource limited intervention.

*Comment* It seems that remote ERP may represent an effective therapy for Tourette’s and chronic tic disorders. However, compliance and engagement may be an issue for this patient group as well as access to appropriate technology and may not be available in less developed countries. In addition, it will be important to understand how this intervention can be integrated within existing treatment pathways, and how remote versus face-to-face ERP compares.

Hollis C, et al. Lancet Psychiatry. 2021 Oct;8(10):871–882.

